# Incidence of infection after early intramedullary nailing of open tibial shaft fractures stabilized with pinless external fixators

**DOI:** 10.4103/0019-5413.43382

**Published:** 2008

**Authors:** Vikas Kulshrestha

**Affiliations:** Department of Orthopaedics, Air Force Hospital, Jorhat - 785 005, Assam, India

**Keywords:** Open tibial fracture, pinless fixator, intramedullary nailing

## Abstract

**Background:**

A major drawback of conventional fixator system is the penetration of fixator pins into the medullary canal. The pins create a direct link between the medullary cavity and outer environment, leading to higher infection rates on conversion to intramedullary nailing. This disadvantage is overcome by the AO pinless fixator, in which the trocar points are clamped onto the outer cortex without penetrating it. This study was designed to evaluate the role of AO pinless fixators in primary stabilization of open diaphyseal tibial fractures that received staged treatment because of delayed presentation or poor general condition. We also analyzed the rate of infection on early conversion to intramedullary nail.

**Materials and Methods:**

This study is a retrospective review of 30 open diaphyseal fractures of tibia, which were managed with primary stabilization with pinless fixator and early exchange nailing. Outcome was evaluated in terms of fracture union and rate of residual infection. The data were compared with that available in the literature.

**Results:**

All the cases were followed up for a period of 2 years. The study includes Gustilo type 1 (n=10), 14 Gustilo type 2 (n=14), and type3 (n=6) cases. 6 cases (20%) had clamp site infection, 2 cases (6.7%) had deep infection, and in 28 cases (93%) the fracture healed and consolidated well.

**Conclusion:**

This study has highlighted the valuable role of pinless external fixator in the management of open tibial fractures in terms of safety and ease of application as well as the advantage of early conversion to intramedullary implant without the risk of deep infection.

## INTRODUCTION

Tibia is the commonest site for open fractures.^1^ These injuries often result in extensive damage to the soft tissue and bone. With high rates of infection and frequent injury to neurovascular structures, they have a high incidence of complications and poor treatment outcome.^2^ Treating these injuries requires experience and judgment.^3^ It remains one of the most challenging problems facing the orthopedic surgeon.^4^ Modern-day management of these injuries has focused on thorough debridement, immediate bony stabilization, and tissue cover to enable early mobility and restoration of optimum function.^5^–^7^

Primary internal fixation in the form of interlocked nailing is being undertaken in most of these fractures.^3^,^8^–^10^ Staged treatment with primary external fixation is preferred only for patients who either present late^11^,^12^ or have multiple injuries precluding immediate intramedullary nailing. All models of external fixators suffer from one critical disadvantage: they perforate the cortex and enter the medullary canal, thus exposing the canal to pin tract infection. Pin tract infection is seen in more than 40% cases.^13^–^17^ Maurer and Gustilo^18^ in their study showed that exchange nailing after external fixation using pin fixator had 25% chance of deep infection, and the same was as high as 71% when nailing was done after there was an evidence of pin tract infection. Most of the studies have shown a high risk of deep infection (more than 20%) when exchange intramedullary nailing is done after external fixation with pin fixators.^19^–^21^ McGraw and Lim^22^ reported 44% deep infection after exchange nailing.

**Figure 1:**
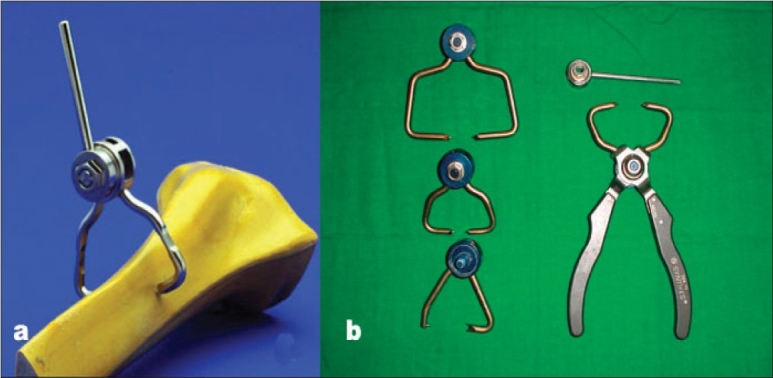
(a) Picture shows unicortical hold of pinless clamps. (b) Picture shows large, small, and asymmetric clamps above downwards on the left; connecting post and locking handle above downwards on right.

The AO pinless external fixator does not violate the intramedullary canal, as the fixator clamps simply rest on the cortical bone without penetrating it [Figure 1]. The medullary canal remains closed, and possible inflammation is restricted to soft tissue. Thus, an early intramedullary nailing is possible.^23^ Pinless fixator rapidly achieves good stabilization of the fracture, while leaving open all options for subsequent treatment.^14^ The primary objective of this study was to asses the role of pinless fixator in primary stabilization of open tibial fractures in patients who underwent staged treatment because of delayed presentation or poor general condition. In addition, we analyzed the incidence of deep infection on conversion to intramedullary nail.

## MATERIALS AND METHODS

We retrospectively reviewed 30 cases of open diaphyseal fractures of the tibia of varying severity that were managed at our institute from 2001 to 2003 with primary stabilization with pinless fixator. All the cases included in the study had a staged treatment (primary external fixation followed by intramedullary nailing) because of either delayed presentation or multiple associated injuries. Cases that had less than two-year follow-up were not included in the study.

**Figure 2:**
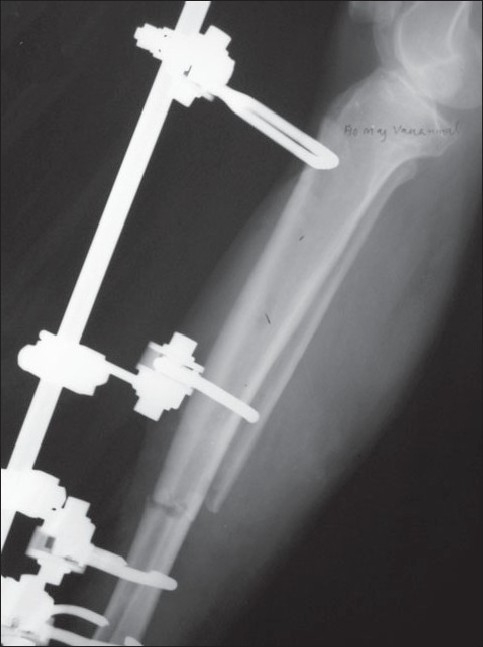
Plain radiograph of fracture of distal third tibia stabilized with pinless fixator. A well-aligned medullary canal with a four-clamp pinless frame can be seen.

**Figure 3:**
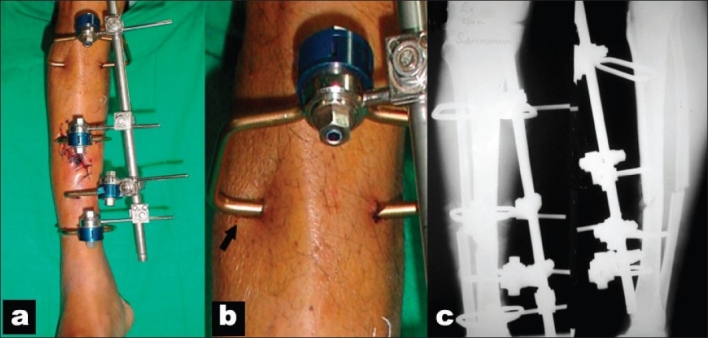
Pictures of pinless fixator used for stabilization of open fracture tibia. (a) An external fixator is in situ for an open fracture of tibia. (b) A close view of the pin site, with limb swelling almost subsided and the pressure on skin around the clamp relieved. (c) Postoperative radiograph shows well aligned fracture stabilized with four-clamp pinless fixator.

All the fractures were classified as per AO fracture classification^24^ for fracture anatomy and Gustilo and Anderson classification^25^,^26^ for nature of open injury. All the fractures selected were of the diaphyseal segment. In all the cases, the pinless fixator was applied within 8 h of hospital admission. The AO pinless external fixator includes three different clamp designs [Figure 2]. For the proximal metaphyseal fixation, large clamps were used; for the midshaft, the asymmetric ones were used; and for distal fixation, the small ones were used. The fixator was applied under spinal anesthesia. Individual clamps were inserted through stab incision with the aid of locking handle. Rocking movements were used to anchor the clamp tips to the outer cortex. This was confirmed by lifting the fragment using the clamp. The rocking movement was avoided during the application of asymmetric clamp. Depending on the geometry of fracture and condition of soft tissues, each main fragment received two clamps. Due care was taken to avoid impaling musculotendinous units by the clamps. This was ensured by making small skin incisions going in till the bone before inserting the clamps. Once the clamps were in position, the fracture was reduced under vision or, if required, under image control, ensuring proper alignment of the medullary canal. This was done to facilitate closed intramedullary nailing subsequently. This was followed by the application of tubular connecting rod and tightening of all screws [Figure 3]. Attempt was made to achieve soft tissue cover by loose closure of soft tissue. However, in two cases of Gustilo type III injuries, adequate soft tissue cover could not be achieved in the first sitting.

In AO type A and B fracture pattern, the pinless fixator ensured reasonable stability permitting early mobilization in the postoperative period. However, in three cases of AO type C fracture pattern, additional below-knee plaster slab was applied to augment stability while transporting the patient after the surgery. In the ward, the slab was removed and the limb was shifted to Bohler Braun splint. In these cases, we permitted only gradual ankle movements in the immediate postoperative period.

**Figure 4:**
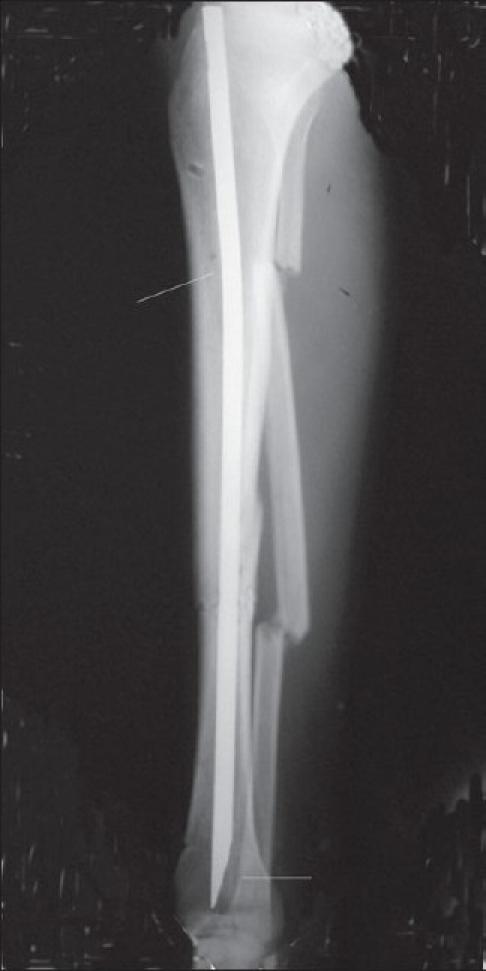
Shows image of the modified K nail used to fix a simple oblique fracture of tibia. The modification in the K nail with anterior bend given in the proximal third of the K nail to compensate for the medullary canal and the oblique leading edge can be seen.

In all cases, intravenous antibiotics were used. In cases with Gustilo type I and II injuries, a cephalosporin was used along with aminoglycoside for 72 h. In all Gustilo type III injuries, the same intravenous antibiotics were given for 5 days along with intravenous metronidazole for the first 3 days. This antibiotic protocol was evolved over a period of time at our institute in consultation with the infection control committee, after a review of available literature^27^–^30^ and study of local antibiotic sensitivity patterns. Adequate period of limb elevation was ensured to combat local swelling. Once swelling subsided, the pressure of clamps over the nearby skin was also relieved, and pin site care was easy [Figure 3b]. Further debridement was done in the operating room at 2- to 5-day intervals as needed, followed by changes of the dressing in the ward as the condition of the wound improved. Meticulous soft tissue care was given with the help of plastic surgeon using biological dressings. Pin site care was given in the form of removal of crust, gentle massage of soft tissue around the pin site, and simple dressing with saline. Non weight bearing ambulation was allowed after stabilization of soft tissue varying from 3 to 7 days in all but three cases of AO type C fracture. Once there were no signs of local wound infection (no swelling, erythema, or discharge), patients were allowed for definitive internal fixation with intramedullary nail. Because of cost constraints in cases of simple AO type A fractures, we used a modified K nail. The conventional K nail was given Herzog bend proximally and distal bevel to negotiate the medullary canal easily. However, since last 4 years, we use only interlock nails. This method was used in seven cases for internal fixation of the fracture [Figure 4]. In all other cases AO type B and C fractures, unreamed interlocking titanium nail (Mathys AG, Bettlach, Switzerland) was used.

A fracture table was used for insertion of unreamed tibial nail (to facilitated distal free hand locking); however, modified K nailing was done on normal table. On the fracture table, the hip was flexed 45°, the knee was flexed 90°, and the thigh was supported with a well-padded crossbar. Traction was not applied, and the foot was just strapped to the foot plate to maintain position [Figure 5A(a)]. The limb was then prepared and draped. A 5-cm long longitudinal incision was made medial to the patellar tendon, and the tendon was retracted laterally. A curved awl was used to make the entrance portal at the midline of the tibia at the level of the fibular head. A nail of proper length and diameter was selected as per template under fluoroscopy and was driven into the medullary canal without reaming. For unreamed tibial nail, the proximal locking jig was attached to the nail before insertion. Care was taken to ensure that the fracture was properly aligned as the nail entered the medullary canal of the distal fragment [Figure 5A(b)]. The fixator was removed after the nail was *in situ*; the distal locking was performed first to enable compression/distraction at the fracture site if required. The nail was statically locked using double proximal and distal locking bolts [Figure 5A(c)].

**Figure 5A:**
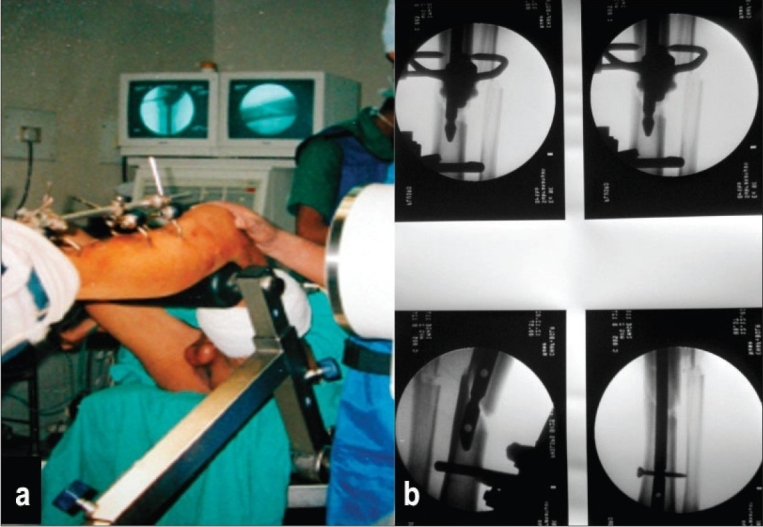
Pictures of closed intramedullary nailing. (a) Shows fractured limb strapped to the foot plate of fracture table being prepared for closed exchange nailing under image intensifier control, with fixator in *situ*. (b) Image intensifier picture shows unreamed tibial nail being inserted by closed technique while pinless fixator maintains reduction.

Following definitive internal fixation, soft tissue condition was evaluated. In most cases, no additional soft tissue procedure was required, as the wound had almost dried up with decrease in limb swelling. However, in six patients, split skin grafting was required, and in two patients of Gustilo 3B fracture, local fasciocutaneous rotation flap cover was required to achieve wound cover. In all cases with interlocked nail *in situ*, partial weight-bearing ambulation was started as tolerated by the patient in immediate postoperative period. In cases with modified K nail *in situ*, partial weight bearing was started 2 weeks postoperatively after the removal of sutures and application of below knee patellar tendon bearing cast.

All cases were closely followed in the postoperative period for evidence of superficial or deep infection at the fracture site including pin-site infection. Cases with local pain, swelling, erythema, and serous discharge (culture positive for pathogen) from the fracture site were labeled as superficial infection and were managed with elevation, drainage, and additional course of antibiotics as dictated by culture sensitivity. Patients were also monitored for evidence of deep infection in the form of wound abscess and systemic features of infection including radiological evidence of periostitis or osteomyelitis. Pin-site infection was considered to be present when there was culture-positive purulent discharge from one or more pin sites when the fixator was removed.

All the cases were followed up for 6 months to 2 years for union and other complications like secondary osteomyelitis and nonunion. The subjective and objective criteria used for establishing union were absence of pain on full weight bearing, lack of swelling, tenderness, or abnormal mobility at the fracture site with radiographic evidence of bridging callus in various phases of consolidation. The injured limb was labeled free of infection in the absence of pain, swelling, or discharge from the fracture site or any of the clamp sites along with the absence of any radiological evidence of osteomyelitis, like sclerosis, osteolysis, and implant loosening.

## RESULTS

The mean age of the patients was 34.9 years (ranged 21 to 60 years) [Table 1]. Most of the fractures (n=16) were wedge type with varying levels of fibular fracture (AO type 42B). Of the remaining cases, eight fractures were simple AO type 42A fractures, and six had complex AO type 42C fracture pattern. On debridement, 14 patients had Gustilo type II injury, 10 patients had Gustilo type I, and 6 patients had Gustilo type III injury and complex fracture anatomy. Average delay in debridement and application of external fixator was 28.4 h (range, 4–72 h) from the time of injury. The delay was due to late presentation, as many cases had to be moved from remote locations. Average delay in intramedullary nailing was 7.9 days. Most of the type 1 and type 2 injuries were taken up within 3–10 days for nailing, and type 3 injuries were taken up 12–14 days after injury. In eight cases (six Gustilo I and two Gustilo II), modified K nailing was done, and in the rest, unreamed interlocked titanium nail (UTN) was used. Few complications which occurred have been listed in Table 2.

**Table 1:** Results of thirty cases of open fracture of the tibia managed with primary stabilization with external fixator

S no.	Age	AO type of fracture	Gustilo type	Delay in external fixation (h)	Delay in nailing (days)	Type of nailing	Wound cover	Secondary procedure	Union (weeks)	Complications
1	21	42-A3.2	GI	8	3	K Nail	Nil	Dynamization	16	Nil
2	42	42-A2.2	GII	6	5	K Nail	Nil	Curettage	24	Nil
3	36	42-B2.2	GII	24	7	UTN*	SSG	Nil	26	Nil
4	35	42-B2.3	GI	32	6	UTN	Nil	Dynamization	22	Nil
5	51	42-B2.3	GII	36	10	UTN	Nil	Nil	22	Nil
6	35	42-A2.2	GI	48	5	K Nail	Nil	Nil	18	Nil
7	33	42-B2.3	GIII A	56	12	UTN	SSG	Curettage	38	Nil
8	39	42-B3.3	GI	60	7	UTN	Nil	Dynamization	20	Nil
9	22	42-B3.2	GII	24	12	UTN	Nil	Nil	22	Nil
10	24	42-C1.2	GIIIA	8	14	UTN	SSG	Dynamization	40	Nil
11	29	42-C2.2	GII	6	10	UTN	Nil	Curettage	36	Nil
12	30	42-A2.2	GI	10	5	K Nail	Nil	Nil	20	Nil
13	31	42-B2.3	GII	24	5	UTN	Nil	Dynamization	22	Nil
14	60	42-C2.2	GIII B	72	12	UTN	Flap	Nil	-	Nonunion
15	32	42-B2.2	GII	12	5	UTN	Nil	Nil	30	Nil
16	22	42-A3.2	GI	10	4	K Nail	Nil	Dynamization	18	Nil
17	43	42-A2.2	GII	4	4	K Nail	Nil	Curettage	22	Nil
18	35	42-B2.2	GII	24	8	UTN	SSG	Nil	28	Nil
19	36	42-B2.3	GI	30	5	UTN	Nil	Dynamization	20	Nil
20	50	42-B2.3	GII	38	11	UTN	Nil	Nil	24	Nil
21	36	42-A2.2	GI	46	4	K Nail	Nil	Nil	16	Nil
22	32	42-B2.3	GIII A	58	13	UTN	SSG	Curettage	40	Nil
23	41	42-B3.3	GI	58	6	UTN	Nil	Dynamization	18	Nil
24	21	42-B3.2	GII	22	13	UTN	Nil	Nil	24	Nil
25	28	42-C1.2	GIII A	8	13	UTN	SSG	Dynamization	38	Nil
26	28	42-C2.2	GII	8	11	UTN	Nil	Curettage	38	Nil
27	31	42-A2.2	GI	12	4	K Nail	Nil	Nil	18	Nil
28	30	42-B2.3	GII	22	6	UTN	Nil	Dynamization	24	Nil
29	61	42-C2.2	GIII B	70	11	UTN	Flap	Nil	-	Nonunion
30	31	42-B2.2	GII	12	6	UTN	Nil	Nil	28	Nil

* Unreamed tibial nail.

**Table 2:** Complications/difficulties encountered in management

S no.	Results	Incidence(%)
1	Delayed union requiring dynamization	10 (33)
2	Superficial soft tissue infection due to clamp	06 (20)
3	Deep infection and infected nonunion	02* (07)
4	Unstable frame prevented early mobilization	03† (10)
5	Secondary cover required	08 (27)

* Both were Gustilo III B injuries with > 10 days delay in exchange nailing.

† All these fractures were AO type 42C.

In six patients (20%), there was a discharge from the clamp site, which was cured by curettage of the outer cortex and oral antibiotics for a short period. This did not affect the result of exchange nailing. In none of these cases, there was an evidence of deep infection or implant loosening. Two patients (6%) with Gustilo IIIB injury developed deep infection around the intramedullary nail 3–4 weeks after nailing. The fracture developed into an infected nonunion, requiring removal of the implant. Both the patients were later treated with ring fixator. Both these cases reported close to 3 days after the injury, as against most of the other cases, which reported within a day of the injury (average 28.4 h). In both these cases, there was more than 10 days of delay in the exchange nailing, and wound cover was achieved with split skin grafting.

In most of the cases (27 out of 30), pinless fixator provided reasonable stability to the fracture, and the patient was able to perform almost full range of knee and ankle movements. However, weight bearing ambulation was not permitted. In three cases (10%), pinless fixator could not ensure reasonable stability, and all these cases had complex fracture anatomy (AO type 42C). In these cases, full range of knee and ankle movements were not permitted, and additional protection was provided in the form of immobilization over Bohler Braun splint. Ambulation was not permitted in all these cases.

Only during the first 24–48 h, while there was significant swelling of the limb, the pin-site care was difficult because there was some pressure of the clamp claws over the surrounding skin. Once the limb swelling subsided, pressure over the surrounding skin was relieved. Subsequently, the pin site care was same as that for any pin fixator. There were no cases of skin necrosis due to clamps.

In all the cases, soft tissue healing was excellent in the postoperative period. Once the initial limb swelling subsided, most of the wounds (73%; 22 out of 30) shrunk in size and healed rapidly without any sign of infection. To achieve complete soft tissue cover, split skin grafting was required in six cases (20%), and local fasciocutaneous rotation flap was required in two cases of Gustilo IIIB fracture (7%). However, these procedures were done after exchange nailing. In no case, there was any evidence of distal neurological deficit.

In 10 cases, during the 12-week postsurgery follow-up, dynamization of the fracture site was required, as there was clinicoradiological features of delayed union in the form of mild pain at the fracture site on weight-bearing and poor bridging callus. Out of the 10 cases, in eight cases the fracture had been fixed with statically locked unreamed nail which were dynamized by removing the distal static locking bolts when the fracture was close to proximal third and the proximal ones when it was close to distal third. In two cases, where modified K nailing was done, dynamization of the fracture was achieved by fibulectomy. In all these cases, the fracture united at 5–6 months from injury. Average time to union was 25.4 weeks [Figure 5B]. In Gustilo type I fractures, the average time to union was 19 weeks, whereas for Gustilo types II and III, it was 26 and 39 weeks, respectively. There were two cases of frank nonunion (7%), both followed by infection of the fracture site and loosening of the implant. There were no cases of malunion or hardware failure.

**Figure 5B:**
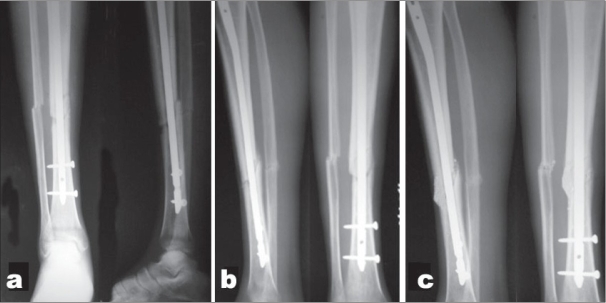
(a) Postoperative radiograph AP and lateral of the fracture showing good reduction and fixation with unreamed tibial nail (UTN) and double distal locking in situ. (b) Radiograph lateral and AP after 12 weeks of surgery, showing ongoing bridging of fracture site and consolidation. (c) Radiograph lateral and AP after 24 weeks of surgery, in which healing is almost complete.

## DISCUSSION

Primary interlocking nail can safely and reproducibly stabilize most low-energy and selected high-energy open fractures of the leg.^3^ However, primary nailing may not be feasible in cases of delayed presentation and polytrauma. These cases require staged reconstructive protocol using external fixation as the primary method of bony stabilization. Review of the available literature shows that use of conventional pin fixators is associated with a high risk of pin tract infection. Hence, exchange intramedullary nailing within 10–12 days is recommended. If exchange nailing is delayed (more than 3 weeks), the rate of infection rises remarkably (20–45%). If there is established pin tract infection, it may even be as high as 71%.^13^,^15^,^18^,^31^–^33^ The AO pinless fixator for the tibia has been designed as a stable, temporary, minimally invasive fixator for severe tibial fractures, ensuring safer conversion to an intramedullary nail.^30^ Early conversion to a more stable implant with significantly reduced incidence of infection is one of the primary goals of this system.^31^ Review of literature did not reveal any study that conclusively established a decrease in the rate of infection following exchange nailing after primary stabilization with pinless fixators.

We received patients from remote locations with multiple injuries. At the time of initial debridement (average delay 28.4 h), primary internal fixation was not possible because of the condition of the wound. There were few cases in which primary internal fixation was ruled out because of associated injuries. With staged treatment using pinless fixator for primary stabilization of the fracture, the overall rate of deep infection was 6%. This result is comparable to that of primary unreamed nailing of these fractures wherein various recent studies^13^,^22^,^23^,^32^,^34^ have revealed infection rates of 5–12% for management of Gustilo types II and III fractures. Pinless fixator is of help during exchange nailing, as it can be used to maintain fracture reduction while exchange intramedullary nailing is performed under image intensifier. This avoids unnecessary manipulation of the limb to achieve reduction, thus preventing further damage to the soft tissue^14^,^31^,^35^–^39^.

Few problems of pinless fixator that have been brought out by some studies are poor stability provided by pinless frame, skin necrosis and sloughing around the pin site, impalement of musculotendinous units including damage to peroneal nerve, and difficulty in performing secondary procedures for skin cover. Winkler *et al*. observed that the arm of the pinless clamp does cause increased pressure on the skin and surrounding soft tissue, which may lead to skin necrosis.^38^ In this study, we saw that once the swelling subsided, there was no pressure on the surrounding soft tissues. During the insertion of clamps, a wider skin incision (instead to stab incisions) was made to avoid pressure on the surrounding tissue. Thomas found that the pinless external fixator endangered important anatomical structures and that safe zones could not always be defined.^23^ Plastic surgical approaches were made more difficult by the pinless fixator, which imposed limitations on local flap design and endangered the arterial perforators that supply them. We did not encounter any difficulty in safe placement of the clamps over the subcutaneous medial surface of the tibia. However, necessary care was taken not to impale the structures on the lateral side by giving larger skin incision and ensuring safe passage of the clamp till the bony surface. Regular wound care and occasional debridement was convenient even with the fixator applied. Procedures like flap cover and split skin grafting were done after exchange nailing. With the fixator *in situ,* it was not possible to do a flap cover; however, a split skin cover could be achieved without difficulty.

The pinless fixator provides enough stability to maintain reduction and allow good range of movements of the adjoining joints.^11^ The pinless fixator is stable enough for temporary fracture fixation of the tibia in a four-clamp, 1-bar construction. Proper application technique (“grab test”, rocking movements) is a prerequisite for stability. Weight-bearing should be avoided and needs a compliant patient.^34^ Reimeger in his study showed that the pinless configurations with small clamps and 1-bar pressure showed stiffness values (as a percentage of the corresponding AO-tubular fixator): 42/36% (steel/titanium clamp) axial stiffness, 61/43% bending stiffness perpendicular to the reference plane, 78/79% bending stiffness parallel to the reference plane, and 90/95% torsional stiffness.^19^ When compared with AO pin fixators, pinless frame is purely a temporary method of stabilizing open tibial shaft fractures, needing early revision to definitive internal fixation. This obviates the need for a very rigid frame that permits weight-bearing and can be kept for a prolonged period of time.

There are few limitations of the study. First, there were no blind observers. Second, although AO classification of fracture pattern is quiet accurate with negligible intraobserver variation, the same cannot be said for Gustilo classification of open injury. Finally, being a retrospective study, proper randomization and comparison with a control cohort who underwent primary stabilization with pin fixator was not possible; hence, the available literature was used to compare the results of this study. The major strength of the study is a well-defined uniform protocol followed for all cases, operated and managed at the same institute by the same surgeon. This excludes many other variables that change from institute to institute and from surgeon to surgeon.

The additional benefits of the system as highlighted in this study have evolved with the increasing use of this system. The advantages of pinless fixation are its simplicity of application, its nonpenetrating clamp, and its use as a reduction aid for conversion to intramedullary nail. This study shows that the use of pinless external fixator, when indicated (delayed presentation/polytrauma situation) for primary stabilization of open tibial shaft fracture, facilitates further management and decreases the rate of infection after intramedullary nailing as seen with the use of pin fixators. However, it cannot be used for definitive fixation of the fracture. It is a valuable addition to the existing AO tubular system.
